# Combined use of bacterial artificial chromosomes-on-beads with karyotype detection improves prenatal diagnosis

**DOI:** 10.1186/s13039-019-0416-6

**Published:** 2019-02-22

**Authors:** Zhengyou Miao, Xia Liu, Furong Hu, Ming Zhang, Pingli Yang, Luming Wang

**Affiliations:** 1 Center of Prenatal Diagnosis, The Maternity and Child Health Care Hospital Affiliated to Jiaxing University, Jiaxing, 314050 Zhejiang Province People’s Republic of China; 2 Center Laboratory Medicine, The First Affiliated Hospital of Jiaxing University, Jiaxing, 314001 Zhejiang Province People’s Republic of China; 3 Hangzhou Biosan Biochemical Technologies Co, Ltd, Hangzhou, China

**Keywords:** Karyotyping, Prenatal diagnosis, Chromosome disorders, Chromosome aberrations

## Abstract

**Background:**

This study evaluated the individual and combined diagnostic performance of the bacterial artificial chromosomes (BACs)-on-Beads (BoBs™) assay and conventional karyotyping for the prenatal detection of chromosomal abnormalities in pregnant women who were 35 or more years-old.

**Method:**

The primary outcome was concordance of any numerical, structural, or submicroscopic chromosomal abnormalities between BoBs™ and conventional karyotyping of amniotic fluid specimens from pregnant women at 17 to 22 weeks gestation.

**Results:**

We examined samples from 4852 pregnant women. BoBs™ indicated that 4708 samples were normal (97.03%), and 144 were abnormal (2.97%); conventional karyotyping indicated that 4656 (95.96%) samples were normal and 196 (4.04%) were abnormal. The combined use of both methods indicated that 4633 of 4852 samples were normal (95.49%) and 219 of 4852 samples (4.51%) were abnormal. The kappa coefficient of the combined test was 0.70, indicating substantial consistency between BoBs™ and conventional karyotyping (95% CI = 0.65–0.76, *P* < 0.001).

**Conclusions:**

Our results indicate that the combined use of BoBs™ and conventional karyotyping detected more fetal abnormalities than either test alone.

## Background

For several decades, traditional Giemsa banding chromosome analysis and prenatal diagnosis using amniocentesis and chorionic villus sampling have had an integral role in clinical workups that aim to prevent neonatal deaths, stillbirths, and pregnancy losses during the first and second trimesters [1]. Approximately 30% of miscarriages result from aneuploidy, and at least 0.3% of newborns have numerical chromosomal abnormalities that can be detected using traditional karyotyping [2–5]. Traditional chromosome analyses enable the detection of large genomic alterations, such as triploid, aneusomy, balanced and unbalanced chromosomal rearrangements of at least 3–5 Mb in size, and mosaicism [6]. For example, traditional screening for trisomy 21 has a detection rate of 80–90% if performed during the first or second trimester, with an average false-positive rate of 5%.

However, new prenatal screening methods are needed for other chromosomal diseases to increase detection rates, and decrease false-positive and false- negative results, so that unnecessary and invasive diagnostic tests are not administered [7, 8]. Several recent studies introduced an array comparative genomic hybridization (aCGH) technique using the bacterial artificial chromosomes (BACs)-on-Beads (BoBs™) technology for the detection of common aneuploidies and specific microdeletion syndromes [1, 9–11]. The BoBs™ assay measures the number of DNA copies at the level of chromosome arm resolution, such as genomic rearrangements and DNA gains or losses, and was designed to detect 9 microdeletion syndromes and the number of chromosome copies, including trisomy 13, 18, 21, X, and Y [12–14]. Several studies have demonstrated that the BoBs™ assay is an accurate, robust, and efficient method for the rapid diagnosis of common aneuploidies and microdeletion syndromes in prenatal samples [15–20]. However, neither BoBs™ nor karyotyping alone is 100% accurate for the detection of fetal chromosomal abnormalities. Thus, testing for fetal chromosomal abnormalities in the cells of amniotic fluid using a combination of chromosome G karyotype analysis and the BoBs™ assay should provide more accurate results [21].

This study compared the individual and combined use of the BoBs™ assay and conventional karyotyping for the prenatal detection of chromosomal abnormalities in the amniotic fluid cells of women who were at least 35 years-old and at 17 to 22 weeks gestation.

## Methods

Amniotic fluid samples were obtained from 4852 pregnant women who were at 17 to 22 weeks of gestation and were patients at a single hospital in China (First Affiliated Hospital of Jiaxing University). This hospital is one of the largest in Jiaxing, and is visited by a large population, including patients from neighboring regions. Thus, this study includes patients from several nearby regions of China. All included pregnant women had at least one of the following indications for invasive prenatal diagnosis: *(i)* advanced maternal age (≥35 years); *(ii)* prenatal serology screening indicating a high risk; *(iii)* abnormal fetal ultrasound screening results; *(iv)* high risk of other suspected chromosomal abnormalities; and *(v)* previous birth to a baby or having a fetus with a chromosomal abnormality or microdeletion syndrome [22]. Prenatal amniotic fluid examination specimens were taken at the study institution between July 17, 2014 and May 25, 2016. All women were fully informed of the study, and signed informed consent documents prior to enrollment. The study was approved by the ethics committee of the First Affiliated Hospital of Jiaxing University (Approval number?).

### Collection of amniotic fluid

Pregnant women undergoing prenatal diagnosis received B-ultrasound-guided trans-abdominal amniocentesis, during which 25–30 mL of amniotic fluid was obtained. A 20 mL aliquot was used for the cell cultures and karyotyping, and a 5–10 mL aliquot was used for the BoBs™ assay.

### BoBs™ technique

The BoBs™ assay was performed using a prenatal chromosome aneuploidy and microdeletion detection test kit (Perkin Elmer, Waltham, MA, USA), according to the manufacturer’s instructions. Genomic DNA from the specimens and reference DNA were first marked with biotin using an enzymatic method. A polymerase chain reaction (PCR) purification test kit was then used to purify the marked genomic DNA. After purification, the mixture of marked genomic DNA and BoBs™ was subjected to single-cell hybridization overnight. The microbeads were washed after hybridization, and were then incubated with the reporter molecule (streptavidin-phycoerythrin), which caused the reporter molecule to bind to the biotin-marked genomic DNA. The assay was performed after the microbeads had been washed again and resuspended.

A Luminex 200 (Austin, TX, USA) flow cytometry instrument was used to measure the fluorescence of DNA bound to the microbeads, and BoBsoft™ analytical software (Perkin Elmer) was used for data analysis. The ratio of specimen fluorescence to reference fluorescence was calculated. According to the manufacturer, a ratio greater than 1.0 indicated the chromosome fragments were repeated and a ratio less than 1.0 indicated a deletion.

### Karyotyping technique

A 20 mL aliquot of amniotic fluid was inoculated into one of two culture vessels, and the fluid was incubated in two different incubators for 7–11 days in BIOAMF-2 culture medium (Biological Industries, Israel). Trypsin digestion was used to harvest fragments. Giemsa staining was used to detect banding, and a fully automated chromosome image analyzer (AI Cytovision, Great Britain) was used to complete the fetal karyotyping. Karyotypes are expressed in accordance with ISCN 2016 [23].

### Statistical analysis

The primary outcome measure was the concordance of any numerical, structural, or submicroscopic chromosomal abnormality between BoBs™ and conventional karyotyping. Categorical data are presented as numbers and percentages. Cohen’s kappa coefficient was determined to compare the results from BoBs™ and conventional karyotyping. A two-sided *P* value below 0.05 was considered significant. All statistical analyses were performed using SAS® software (version 9.4, SAS Inc., Cary, NC, USA).

## Results

We excluded 71 samples because of missing information on the fetus sex, and ultimately enrolled and analyzed 4852 diagnostic samples (Table 1). BoBs™ indicated that 4708 (97.03%) of the samples were normal and 144 (2.97%) were abnormal; conventional karyotyping indicated that 4656 (95.96%) of the samples were normal and 196 (4.04%) were abnormal. A combined use of both methods indicated that 4633 (95.49%) of the samples were normal and 219 (4.51%) were abnormal. Thus, the combined use of BoBs™ and karyotyping detected more fetal abnormalities than BoBs™ alone or karyotyping alone.

**Table 1:** Detection of abnormalities in 4852 fetuses by use of BACs-on-Beads (BoBs™) alone, conventional karyotyping alone, and their combined use

Prenatal BoBs™ (*n*, %)	Conventional karyotyping (*n*, %)	Combined use of BoBs™ and karyotyping (*n*, %)
Normal (4708, 97.03%)	Normal (4656, 95.96%)	Normal (4633, 95.49%)
Abnormal (144, 2.97%)	Abnormal (196, 4.04%)	Abnormal (219, 4.51%)

BoBs™: Bacterial artificial chromosome (BACs)-on-Beads examination

A comparison of BoBs™ with conventional karyotyping had a kappa coefficient of 0.70 (*P* < 0.001, 95% confidence interval [CI]: 0.65–0.76); a comparison of BoBs™ with the combined use of BoBs™ and conventional karyotyping had a kappa coefficient of 0.79 (95% CI: 0.74–0.83, *P* < 0.001); and a comparison of conventional karyotyping with the combined use of BoBs™ and conventional karyotyping had a kappa coefficient of 0.94 (95% CI: 0.92–0.96, *P* < 0.001). These results indicate substantial consistency between the combined use of BoBs™ and conventional karyotyping, BoBs™ alone, and karyotyping alone.

BoBs™ and conventional karyotyping had the same rates for detecting the most common abnormalities: trisomy 13 (0.02%), trisomy 18 (0.49%), trisomy 21 (1.69%), Turner syndrome (0.08%), and Klinefelter syndrome (0.06%) (Table 2). BoBs™ also screens for 9 microdeletion syndromes (Wolf-Hirschhorn syndrome, *n* = 0; Cri du Chat syndrome, n = 0; Williams-Beuren syndrome, *n* = 1; Langer-Giedion syndrome, n = 0; Prader-Willi/Angelman syndrome, n = 0; Miller-Dieker syndrome, n = 0; Smith-Magenis syndrome, n = 0; DiGeorge syndrome, *n* = 2; and DiGeorge II syndrome, n = 0). However, only BoBs™, not conventional karyotyping, detected DiGeorge syndrome (0.04%) and Williams-Beuren syndrome (0.02%), and the subsequent fetal outcomes were abortion. However, inconsistent findings between BoBs™ and conventional karyotyping, the fetal outcomes were found in the case of Turner syndrome and the others, or both.

**Table 2:** Fetal abnormalities detected using BACs-on-Beads (BoBs™) alone and conventional karyotyping alone

Prenatal BoBs™ (*n*, %)	Conventional karyotyping (*n*, %)	Gender	Fetal outcome	Number
Trisomy 13 (1, 0.02%)	Abnormal (1, 0.02%)	Male	Abortion	1
Trisomy 18 (24, 0.49%)	Abnormal (24, 0.49%)	Female	Abortion	15
		Male	Abortion	9
Trisomy 21 (82, 1.69%)	Abnormal (82, 1.69%)	Female	Abortion	42
		Male	Abortion	39
DiGeorge syndrome (2, 0.04%)	Normal (2, 0.04%)	Male	Abortion	2
Williams–Beuren syndrome (1, 0.02%)	Normal (1, 0.02%)	Female	Abortion	1
Turner syndrome (4, 0.08%)	Abnormal (4, 0.08%)	Female	Normal	1
		Female	Abortion	3
Klinefelter’s syndrome (3, 0.06%)	Abnormal (3, 0.06%)	Male	Abortion	3
Others^a^ (27, 0.57%)	Normal (20, 0.41%)	Female	Normal	7
		Female	Abortion	1
		Male	Normal	10
		Male	Abortion	2
	Abnormal (7, 0.15%)	Female	Abortion	5
		Male	Abortion	2

BoBs™: Bacterial artificial chromosome (BACs)-on-Beads examination

-: Sex data missing

^a^15q11, 18p11, 22q11.2, 7q11.2, Xp22.31, Yq11 chromosomal region enrich, Xp21-p22, Xp22.31 chromosomal region deletion, 47XXX, 47XYY

Only one woman had twins. Because of the special nature of twins and sampling difficulties with twins, diagnosis using amniocentesis is not usually recommended for these women in clinical practice, unless there is a clear medical indication or the woman strongly requests this procedure.

## Discussion

To our knowledge, this was the first study of its type in China that collected data from a large number of patients (> 4800). After excluding 71 samples because of missing information on fetus sex, we analyzed a total of 4852 diagnostic samples. The BoBs™ assay indicated that 4708 samples (97.03%) were normal and 144 samples were abnormal (2.97%); conventional karyotyping indicated that 4633 samples (95.49%) were normal and 219 samples (4.51%) were abnormal; and the combined use of both methods indicated that 4633 samples (95.49%) were normal and 219 samples (4.51%) were abnormal. Therefore, the combined use of the BoBs™ assay with karyotyping improves the prenatal detection of fetal abnormalities.

### BoBs™ is better than karyotyping in detecting copy number variations

BoBs™ uses a unique assay probe, in contrast to karyotyping, which examines the entire chromosomal structure, and thus provides accurate assessments of changes in certain micro-areas. Furthermore, the sensitivity of BoBs™ at detecting tiny abnormalities of this sort cannot be achieved by any current karyotyping methods, all of which focus on detecting chromosome-level abnormalities [15]. In particular, BoBs™ detected 25 cases of chromosomal microduplications and microdeletions. However, karyotyping detected only two of these abnormalities, based on chromosomal structural abnormalities.

Other studies have demonstrated similar results (Table 3). For example, Leung et al. [21] performed a retrospective study of 2053 prenatal cases (1421 uncultured chorionic villus samples, 616 amniotic fluid samples, and 16 other clinical samples), and identified 132 non-mosaic cases of trisomy 21, 18, and 13 by use of traditional karyotyping and by use of the BoBs™ assay. However, one case of trisomy 18 that was identified by karyotyping and BoBs™ was determined inconclusive for chromosome 18 based on quantitative fluorescence (QF) PCR because of a borderline abnormal ratio of 1.3 to 1.6 [21]. Leung et al. [21] concluded that traditional karyotyping was 100% concordant with the BoBs™ assay for all non-mosaic cases of trisomy 21, 18, and 13.

**Table 3:** Summary of the present study and previous studies that used BoBs™ and other diagnostic techniques

Study	Results
Present Study	Prenatal BoBs™: Normal (4708, 97.03%); abnormal (144, 2.97%)
	Conventional karyotyping: Normal (4656, 95.96%); abnormal (196, 4.04%)
	Combined use of BoBs™ and karyotyping: Normal (4633, 95.49%); abnormal (219, 4.51%)
	Combined use of BoBs™ and karyotyping detected more abnormalities (4.51%) than BoBs™ alone (2.97%) or karyotyping alone (4.04%)
Leung et al. [21]	Traditional karyotyping and BoBs™: 2053 prenatal cases (1421 uncultured chorionic villus samples, 616 amniotic fluid samples, 16 other clinical samples)
	Traditional karyotyping: 100% concordance with BoBs™ for all non-mosaic cases involving trisomy 21, 18, and 13
Saldarriaga et al. [24]	BAC aCGH plus karyotyping vs. karyotyping or BAC aCGH alone: 9974 pregnant patients
	aCGH: higher sensitivity (94.5% vs. 67.3%) and lower false-negative rate (4.5% vs. 33%) than karyotyping
	No significant difference in false positives for aCGH and karyotyping (1.3% vs. 1%)
Perez-Duran et al. [25]	BoBs™: 50 samples from spontaneous abortions before 20 weeks gestation
	32% of samples had chromosomal abnormalities, 50% of which were the most common chromosomal abnormalities (Down syndrome, Turner syndrome, and trisomy 13)
Vialard et al. [11, 26]	BoBs™ (first study [11]): 408 samples and prospective testing of 212 consecutive samples: no false-positive results; no triploids; mosaic conditions at 20–30%; high predictive value (1 of 1700); high sensitivity (> 98%) and specificity (> 99%); false-negative rate below 2%
	BoBs™ (second study [25]): 1653 prenatal samples: failure rate of 3.3%; overall detection rate of approximately 1 in 10. Detected abnormalities: 85% common aneuploidies; 11 duplications and microdeletions, with overall microdeletion and microduplication rate of 1 in 145
Choy et al. [9]	BoBs™ and karyotyping: 2153 samples
	BoBs™ found 6 microdeletion syndromes, including DiGeorge syndrome, that karyotyping did not detect
	BoBs™ sensitivity was 96.7% and specificity was 100%
	Karyotyping detected 15 (0.7%) cases with major chromosomal abnormalities; BoBs™ detected only 8 (53.3%) of these 15 cases
Garcia-Herraro et al. [28]	BoBs™ combined with karyotyping: 364 prenatal samples; 309 amniotic fluid samples and 35 chorionic villus samples were normal
	Concordance rate of 98.51% between BoBs™ and conventional karyotyping
	3 of 5 samples without agreement had chromosomal abnormalities not detected by BoBs™ (2 Robertsonian translocations, 1 reciprocal translocation and 2 with polymorphisms)
Grati et al. [15]	BoBs™ plus karyotyping: 9648 samples
	Overall incidence rate of 0.7% for cryptic imbalances
	BoBs™ had low a priori risk of approximately 0.3%
Rosenfeld et al. [19]	BoBs™ and karyotyping: 2940 samples
	7.9% aneuploidies and 0.45% partial chromosomal abnormalities
	Combined with karyotyping, additional detection of 1 in 745 for low risk cases (e.g. normal ultrasound and isolated ultrasound marker and increased nuchal measurements), and 1 in 165 for fetal structural or growth abnormalities
Rosenfeld et al. [29]	aCGH compared with other traditional analyses: 535 fetal demise samples
	aCGH detected significant clinical abnormalities in 12.8% of samples characterized as normal or unknown karyotypes
	Normal karyotype subset: significant clinical abnormalities in 6.9% (20 of 288); 107 samples examined by aCGH and SNP: SNP detected significant clinical abnormalities in 7 cases (7.5%)
	aCGH did not provide fetal results for 8.3% (20 cases) because of poor DNA quality and maternal cell contamination

A meta-analysis of five studies of 9974 pregnant patients in several countries (United States, Israel, Italy, and Taiwan) compared BAC aCGH plus karyotyping with karyotyping or BAC aCGH alone [24]. The results indicated that relative to karyotyping, aCGH had higher sensitivity (94.5% vs. 67.3%) and a lower false-negative rate (4.5% vs. 33%), but a similar rate of false positives (1.3% vs. 1%).

Another study in Mexico examined 50 samples obtained from spontaneous abortions that occurred prior to 20 weeks of gestation using the BoBs™ assay [25]. The results indicated that 32% of the samples had a chromosomal abnormality, and half of these abnormalities were among the most common types of chromosomal abnormalities (Down syndrome, Turner syndrome, and trisomy 13 [one case]) [25].

Vialard et al. [11, 26] conducted two studies in Europe using BoBs™ to detect aneuploidies and microdeletions. The first study [11] was a retrospective analysis of 408 samples with prospective testing of 212 consecutive samples. They had no false-positive results, no triploidies; mosaic conditions at 20–30%; a predictive value of 1 in 1700, a sensitivity greater than 98%, a specificity greater than 99%, and a false-negative rate below 2%. The second study [26] assessed 1653 prenatal samples using BoBs™, and had a failure rate of 3.3%, with an overall detection rate of approximately 1 in 10. Among the detected abnormalities, 85% were common aneuploidies (11 duplications and microdeletions), indicating an overall microdeletion and microduplication rate of 1 in 145.

### Karyotyping is better than BoBs™ in detecting chromosomal structural abnormalities

Karyotyping is considered the gold standard method for detecting variations of chromosomal structure [27]. In the present study, BoBs™ detected none of the 76 cases of chromosomal structural abnormalities or variations, all of which were clearly identified by karyotyping.

In addition, karyotyping can detect the ratio of mosaicism, which is difficult with the BoBs™ assay. In particular, a recent study by Choy et al. [9] in Hong Kong demonstrated that BoBs™ was similar to karyotyping in the detection of trisomy 13, trisomy 18, trisomy 21, and sex chromosome aneuploidy among 2153 archived samples. Choy et al. [9] also determined that BoBs™ detected 6 microdeletion syndromes, including DiGeorge syndrome (4 cases), that were not detected by karyotyping. These authors reported that BoBs™ had a sensitivity of 96.7% and a specificity of 100% [9]. However, they also found that karyotyping detected 15 (0.7%) cases that had major chromosomal abnormalities, including structural abnormalities of chromosome 13, 18, and X, but that BoBs™ only detected 8 (53.3%) of these 15 cases (8 of the 10 with targeted chromosomal loci) on chromosome 4, 5, 13, 18, 22, and X. BoBs™ was unable to detect a case of ring chromosomes on 15 and 22, even though the BoBs™ assay was designed to detect certain regions on these chromosomes [9]. Further, Choy et al. detected 7 cases of mosaicism on chromosomes 2, 7, 8, 15, 16, and 22 by karyotyping [9]; the BoBs™ assay does not target chromosome 2 or 16, but it did detect 2 of the other 5 cases of mosaicism in other chromosomes.

### Use of both methods improves accuracy and detection

Our results from the combined use of both methods differed in 5 cases: 3 cases of Robertsonian translocations (chromosome 21 isochromosome for long arm); 1 case of a marker chromosome (BoBs™ indicated a microduplication); and 1 case of 46,X,i(X)(q10) with one X isochromosome, in which the tenth gene on the long branch was triploid or haploid (BoBs™ indicated microdeletions). This indicates that the combined use of both methods provides more accurate detection of abnormalities in chromosome number, copy number, and chromosome structure [28].

A previous study reported similar results regarding chromosomal abnormalities, such as Robertsonian translocations [28]. These Spanish researchers used a BoBs™ assay combined with karyotyping to test 364 prenatal samples, and found that 309 amniotic fluid samples and 35 chorionic villus samples were normal. The concordance rate between the BoBs™ assay and conventional karyotyping was 98.51%; 3 of the 5 samples with discordant results had chromosomal abnormalities that were undetected by the BoBs™ assay (2 Robertsonian translocations and 1 reciprocal translocation), and the other 2 samples had polymorphisms.

Another study prospectively examined 9648 prenatal samples from several laboratories worldwide using karyotyping plus the BoBs™ assay [15]. The overall incidence rate of cryptic imbalances was 0.7%, most of which were in the critical region for DiGeorge syndrome, and the added yield of BoBs™ for patient populations with a low a priori risk was approximately 0.3%.

A study by Rosenfeld et al. [19] in the United States examined 2940 prenatal samples using a quick BoBs™ assay, in which 89% of the results were obtained within 1 day. There were 7.9% aneuploidies and 0.45% partial chromosomal abnormalities. When combined with karyotyping, these researchers detected 1 of 745 cases that had low risk (such as a normal ultrasound or isolated ultrasound marker and increased nuchal measurements) and 1 of 165 cases that had fetal structural or growth abnormalities [19].

### Other aCGH methods for detecting prenatal chromosomal abnormalities

A recent study by Rosenfeld et al. [29] used aCGH with other traditional analyses to assess 535 fetal demise samples. This method was successful for 515 samples, 16 of which had known karyotype abnormalities that were excluded from the analysis, some of which were examined by single nucleotide polymorphism analyses. There were significant clinical abnormalities in 12.8% (64 of 499) of the samples that were characterized as normal or unknown karyotypes [29]. Among the normal karyotypes, significant clinical abnormalities were present in 6.9% (20 of 288) of the samples. They examined 107 samples with aCGH and SNP, and SNP detected significant clinical abnormalities in 7 cases (7.5%), such as female triploidy [29]. However, aCGH did not provide results for 8.3% of the samples (20 cases) because of poor DNA quality and maternal cell contamination [29]. Moreover, they did not obtain karyotype results for 21 cases, although aCGH provided results for all of these 21 cases [29]. Lastly, many of the significant clinical abnormalities they detected with aCGH were under the approximate 10-Mb resolution of karyotyping [29].

Gullotta et al. [30] found that aCGH cannot detect balanced rearrangements, including reciprocal and Robertsonian translocations and inversions, but can identify changes in DNA copy numbers concomitantly at numerous discrete loci. Further, they used aCGH consisting of 167 genomic clones (corresponding to 34 chromosomal regions frequently seen in microdeletions and microduplications) and 126 subtelomeric clones, and demonstrated agreement of all aCGH and karyotyping, DNA, and fluorescence in situ hybridization (FISH) results [30].

Other methods, such as FISH and quantitative fluorescence (QF)-PCR, can also be used for clinical prenatal diagnosis. In the United Kingdom, Caine et al. [31] compared karyotyping by FISH and PCR. They identified 3081 abnormal karyotypes in 98,166 amniotic fluid samples, but FISH or PCR detected only 2075 (67%) of them. In addition, FISH or PCR only detected 1157 abnormal samples (78%) among 1484 abnormal karyotypes from 13,344 chorionic villus samples [31]. Sato et al. [32] examined 79 embryos by probing chromosomes 13, 18, 21, X, and Y using QF-PCR for 151 blastomeres and FISH for 145 blastomeres. They found that FISH analyses could only be performed on 135 blastomeres (93%), and QF-PCR analyses could only be performed on 117 blastomeres (77%), so only identified 20 embryos (31%) as abnormal.

BoBs™ has several advantages over FISH, in that detection results can be obtained within 24 h; culturing, coverslips, and a microscope are not required; a smaller sample (approximately 150–240 ng) can be used; and the data analysis is simpler and provides more information on more diseases [33]. BoBs™ also provides high throughput detection, with simultaneous assays of 92 samples; data are obtained automatically; and high accuracy detection of a single sample requires only 40 s to 2 min [33]. In addition, BoBs™ can assess 4–8 targets to determine the presence of a mutation or disease, a result that would require multiple FISH assessments [33]. Compared with FISH, a BoBs™ assessment requires fewer skills and is less expensive [33].

Gekas et al. [34] studied 100,948 pregnancies for detection of Down syndrome, and determined that the most inexpensive method was QF-PCR, with a cost-effectiveness ratio of $24,084 for each detected case, followed by karyotyping with a cost-effectiveness ratio of $27,898. The incremental cost-effectiveness ratio to identify chromosomal abnormalities missed by rapid aneuploidy diagnosis (QF-PCR and FISH) was $66,608 for each chromosomal abnormality. Analyses were possible on 135 blastomeres (93%) by FISH and 117 blastomeres (77%) by QF-PCR. For the 65 embryos that could be analyzed by both methods, 20 embryos (31%) were abnormal.

Although the present study provided evidence that the combined use of karyotyping and BoBs™ greatly improved the detection of chromosomal disorders, there were some limitations. Neither method had a detection rate of 100%, so it is possible that a small number of genetic disorders or chromosomal microdeletions remained undetected. However, given that most other methods can be time-consuming and expensive, other approaches may not be acceptable to women who had spontaneous abortions. Because collection of amniotic fluid entails a certain degree of risk, some pregnant women may refuse this procedure. As a result, our study samples may not have been representative of the whole population. BoBs™ can detect chromosome mosaicism, which is dependent on the proportion of abnormal cells in a sample and the type of abnormality. However, its sensitivity varies for different types of chromosome mosaicism [35]. Among the 4852 samples we examined, karyotype analysis identified three cases of chromosome mosaicisms. The karyotype for the first case was 46,XN,t(12;14)(p11;p12) [2]/46,XN [15], which was not detected by BoBs™. The karyotype for the second case was 45,X [17]/46,X,+mar [13], which BoBs™ detected as 45,X and the karyotype for the third case was mos 45,X [20]/46,XY [2], which BoBs™ also identified as 45,X.

Another potential limitation of our study was the presence of maternal cell contamination. We initially examined the amniotic fluid samples without any visual magnification. If a sample was red, we considered it likely to have been contaminated with red blood cells (presumably maternal), and did not analyze it using BoBs™. In addition, we also excluded samples if an amniotic fluid sample was not obviously red, but a layer of red blood cells appeared after centrifugation. Lastly, we only confirmed the positive results of BoBs™ by chromosomal microarray analysis (data not shown). Future studies should confirm all microdeletions and microduplications detected by BoBs™ using a chromosomal microarray, including false positives.

## Conclusion

BoBs™ has several advantages for prenatal diagnosis, including high accuracy, speed, low initial sample volume, high success rate, easy implementation, and the ability to detect small fragment abnormalities. However, karyotyping can accurately detect many types of chromosomal structural abnormalities and variations that are missed by BoBs™. Thus, these two methods are complementary, and their combined use improves the detection and accuracy of prenatal diagnoses.

## Abbreviations

aCGH: array comparative genomic hybridization
BoBs™: bacterial artificial chromosomes (BACs)-on-Beads (BoBs™)
FISH: fluorescence in situ hybridization
QF-PCR: quantitative fluorescence polymerase chain reaction
